# Editorial: Plant artificial chromosomes: progress and perspectives

**DOI:** 10.3389/fpls.2023.1290386

**Published:** 2023-09-26

**Authors:** Chunhui Xu, James A. Birchler

**Affiliations:** ^1^ Key Laboratory of Plant Development and Environmental Adaptation Biology, Ministry of Education, School of Life Sciences, Shandong University, Qingdao, China; ^2^ Division of Biological Sciences, University of Missouri at Columbia, Columbia, MO, United States

**Keywords:** plant artificial chromosome, site specific recombination, pBTR vectors, synthetic biology, genetic transformation

Plant artificial chromosomes (PACs) are small chromosomes that have been engineered to contain desired genes. Their potential advantage when they reach their full potential is that they will be independent of other chromosomes in the karyotypic complement and will not exhibit linkage drag carrying potential detrimental alleles when introgressed to new varieties. Further, the ability to stack genes on these independent chromosomes will facilitate such efforts as opposed to combining multiple transgenes in different places in the genome, which rapidly become unmanageable as the numbers increase. Using artificial chromosomes in concert with gene editing approaches has the potential to design and manipulate genomes for many applications.

Given the epigenetic nature of centromeres in plants, which precludes assembly of artificial chromosomes as performed originally in yeast, the first generation of plant artificial chromosomes was produced via telomere-mediated chromosomal truncation (Yu et al., 2007). This approach has subsequently been used to truncate chromosomes in a variety of plant species (Birchler and Swyers, 2020). Potential applications and potential additional developments with minichromosomes have been summarized (Birchler, 2014; Birchler, 2015). This Research Topic provides an expanded collection of approaches that could enhance the utility of plant artificial chromosomes.

As noted, plant artificial chromosomes are considered as an excellent platform to carry multiple genes. This is especially important for polygenic traits. To work as such a platform, a system to continuously stack new genes needs to be established. Professor David Ow’s group has created a site specific gene stacking system *in planta* based on Bxb1 mediated recombination with Cre recombinase being used to remove the sequences not required (Hou et al., 2014). On this topic, Prof. Ow’s group made progress on this system (Yin et al. 2022). Using Cre-lox mediated recombination, the authors showed that transgenes flanked by lox sites can translocate between chromosomes. This system could be used to transfer elite traits from the transgenic plants to the cultivars that are difficult to be transformed and at the same time linkage drag could be avoided.

As gene stacking *in planta* for PACs has not been realized to date, an alternative approach is to use constructs that carrying several genes in the transformation. On this topic, Wang et al. (2022) has developed a set of binary vectors, named pBTR, that can be used to carry multiple genes using the Golden Gate cloning method. The vectors were successfully used in the transformation of tomato and soybean via *Agrobacterium rhizogenes*-mediated transformation. Thus, pBTR vectors could be applied when the traits of crops encoded by multiple genes are to be improved or created through genetic transformation.

As a synthesized platform, expression of the genes on artificial chromosomes needs to be regulated properly. On this topic, Gomide et al., (2022) reviewed the strategy of using biocircuits to facilitate the controlled expression of genes on synthetic chromosomes. In this review, the authors introduced the history and achievements of synthetic biology of plants and algae, the parts and assembly tool kits that have been created, and the computational programs that could be utilized to design the genetic circuits and the strategies to control the gene expression. The major challenges were also discussed.

Although attempts to create PACs have succeeded, a number of barriers need to be overcome before application. To address the related issues, Kan et al., (2022) reviewed the methods for developing PACs, the progress that has been achieved, the factors involved in the formation of PACs, the strategy in stacking exogenous genes, and the limitations to be overcome in the application of PACs.

## Author contributions

CX: Writing – original draft. JB: Writing – original draft, Writing – review & editing.
